# Physiological and Pathological Aging Affects Chromatin Dynamics, Structure and Function at the Nuclear Edge

**DOI:** 10.3389/fgene.2016.00153

**Published:** 2016-08-23

**Authors:** Jérôme D. Robin, Frédérique Magdinier

**Affiliations:** ^1^IRCAN, CNRS UMR 7284/INSERM U1081, Faculté de MédecineNice, France; ^2^Aix Marseille Université, INSERM, GMGF, UMRS_910Marseille, France

**Keywords:** Lamins, premature aging, nuclear topology, epigenetics, telomeres

## Abstract

Lamins are intermediate filaments that form a complex meshwork at the inner nuclear membrane. Mammalian cells express two types of Lamins, Lamins A/C and Lamins B, encoded by three different genes, *LMNA*, *LMNB1*, and *LMNB2*. Mutations in the *LMNA* gene are associated with a group of phenotypically diverse diseases referred to as laminopathies. Lamins interact with a large number of binding partners including proteins of the nuclear envelope but also chromatin-associated factors. Lamins not only constitute a scaffold for nuclear shape, rigidity and resistance to stress but also contribute to the organization of chromatin and chromosomal domains. We will discuss here the impact of A-type Lamins loss on alterations of chromatin organization and formation of chromatin domains and how disorganization of the lamina contributes to the patho-physiology of premature aging syndromes.

## Introduction

In interphase eukaryotic cells, the nucleus appears as a heterogeneous compartment filed with chromatin. Chromatin structure can be functionnaly divided into heterochromatin (condensed and non-active) and euchromatin (less condensed and active). Chromatin dynamics involves a number of proteins such as DNA binding factors, enzymes involved in histone marks deposition or reading, nucleosome sliding, eviction or replacement and molecules regulating DNA methylation.

Several nuclear substructures can be visualized using phase contrast microscopy and a highly complex organization is observable by fluorescence microscopy. Most of the nuclear substructures, including the nuclear pore complex (NPC), the nuclear lamina (NL), and more than 10 different types of nuclear bodies have been characterized (Spector, 2006).

The nucleus is delimited by the nuclear envelope (NE) that lies at the interface between this compartment and the cytoplasm, segregates the DNA from the cytoplasm and allows communication between these two compartments. The NE consists of three intimately linked substructures: the nuclear membranes, the NL and the NPCs. The outer nuclear membrane (ONM) is continuous with the endoplasmic reticulum and shares some features with this cytoplasmic organelle. The NE is interrupted by the nuclear pores allowing connections between the ONM and inner nuclear menbranes (INM), but more importantly nucleocytoplasmic transport. The INM is composed of Lamins and specific integrated proteins called nuclear envelope transmembrane proteins (NETs). To date, more than 90 NETs have been identified, but only a small subset is fully characterized (Chen et al., 2006; Schirmer and Foisner, 2007; Srsen et al., 2011).

**FIGURE 1:**
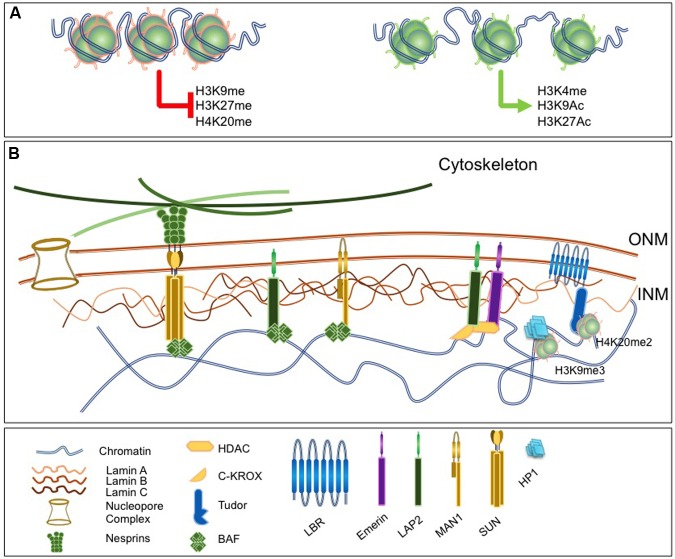
**Lamins as a nuclear scaffold.** **(A)** The nuclear lamina (NL) interacts with modified histones. Left, histones marks associated with constitutive or facultative heterochromatin and involved in chromatin condensation. Right, histone marks associated with euchromatin and open chromatin region. **(B)** The nucleus is delimited by the nuclear envelope (NE) that consists in three substructures, the outer and inner nuclear membranes [outer nuclear membrane (ONM); inner nuclear menbranes (INM)], the nuclear pore and the NL. The NL is a filamentous network of A- and B-type Lamins that ensures nuclear functions and involves a number of lamina-associated proteins such as Barrier to Autointegration Factor (BAF), LAP proteins, Lamin B Receptor (LBR) or Emerin. Proteins of the LINC complex such as Nesprins and Sun proteins extend through the nucleoplasm, associate with microtubules and ensure the continuity between the nucleus and the cytoskeleton. lamina components interact with chromatin and a number of chromatin-associated factors.

At the INM, the lamina constitutes the major protein fraction that resists to detergent-high salt extraction (Aaronson and Blobel, 1975). Lamins, the principal components of this fraction belong to the intermediate filament (IF) supergene family (Aebi et al., 1986; Fisher et al., 1986; Goldman et al., 1986) and form a complex meshwork at the INM. Lamins exist in all metazoans, but are not found in plants and unicellular eukaryotes. In *Caenorhabditis elegans*, there is only one Lamin protein (Riemer et al., 1993; Lyakhovetsky and Gruenbaum, 2014), but in mammals the different isoforms are classified as A- and B-types according to their sequence homologies. B-type Lamins are farnesylated and expressed at the earliest stages of development and later on, whereas A-type Lamins are soluble and their expression seems related to cell differentiation (Dechat et al., 2008). Lamins A/C have a large number of binding partners including other nuclear Lamins, INM proteins as well as chromatin-associated and DNA binding proteins (**Figure 1**). By providing a strong mechanical scaffold for stable nuclear organization, Lamins play different structural roles such as the maintenance of the nuclear shape, rigidity and resistance to mechanical stress (Gruenbaum and Foisner, 2015). In addition, Lamins and associated proteins regulate chromatin by modulating and maintaining heterochromatin and chromosomal domains through interactions with chromatin binding and transcription factors (**Figure 1**). Lamins also contribute to the maintenance of genome integrity through the recruitment of the DNA damage response machinery (Gonzalo, 2014; Gonzalo and Kreienkamp, 2015).

Mutations in A-type Lamins are associated with a group of phenotypically diverse diseases referred to as laminopathies including Hutchinson–Gilford Progeria (HGPS), Emery–Dreifuss Muscular Dystrophy (EDMD), Limb-Girdle Muscular Dystrophy (LGMD), Familial Partial Lipodystrophy (FPLD), Acro-Mandibular Dysplasia (MAD), Charcot-Marie-Tooth, type B (CMT2B) or Restrictive Dermopathy (RD) while Lamin B is mutated in adult-onset demyelinating leukodystrophy (ADLD) [reviewed in Burke and Stewart (2014) and Camozzi et al. (2014)]. The broad impact of A-type Lamins loss on alterations of chromatin organization and formation of chromatin domains will be detailed here with a particular focus on the consequences in progeroid syndromes (Cau et al., 2014).

## Lamin Mutations and Premature Aging Syndromes

Premature aging or progeroid syndromes are a group of rare diseases characterized by clinical features of accelerated aging such as hair loss, skin tightness, cardiovascular diseases, osteoporosis, lipodystrophy, and reduced lifespan. Hence, progeroid syndromes recapitulate clinical features of normal aging but in a precocious manner and their underlying mechanisms may vary depending on the gene or pathways involved. We will focus here on progeroid syndromes associated with mutations in genes encoding components of the NE (Lopez-Otin et al., 2013; Cau et al., 2014; Kennedy et al., 2014).

Among them, HGPS is the most prevalent accelerated-aging syndrome. This disease is linked to a heterozygous mutation in the *LMNA* gene (G608G) encoding A-type Lamins (De Sandre-Giovannoli et al., 2003; Eriksson et al., 2003). Mature Lamin A requires if the nuclear periphery (NP) is generally associated posttranslational modification and cleavage of pre-Lamin A to become mature. The G608G mutation activates a cryptic splicing site that results in the deletion of 50 amino acids at the C terminus of pre-Lamin A, leading to production and accumulation in the nucleoplasm of a toxic protein called progerin; utlimately disrupting the integrity of the NE detectable as nuclear shape distorsions and blebbings (De Sandre-Giovannoli et al., 2003; Eriksson et al., 2003). Other *LMNA* mutations are associated with atypical progeroid syndromes, not systematically associated with progerin accumulation (Fukuchi et al., 2004; Moulson et al., 2007; Doubaj et al., 2012; Barthelemy et al., 2015) or MAD (Novelli et al., 2002). Recessive mutations in the *ZMPSTE24* gene, encoding the Zinc Metallopeptidase STE24 that cleaves the prenylated and carboxy methylated 15-amino acid tail from the C-terminus of pre-Lamin A, are also associated with diseases that share features of accelerated aging such as MAD (Agarwal et al., 2003),or RD (Navarro et al., 2014). Mutation in the *BANF1* (Barrier to Autointegration Factor 1) gene which encodes the BAF1 Lamin-associated protein are linked to the Nestor-Guillermo progeria syndrome (NGPS) clinically resembling HGPS (Puente et al., 2011). Interestingly, progerin also accumulates during physiological aging and correlates with chromatin changes reinforcing the parallel between physiological aging and progeroid syndromes (Scaffidi and Misteli, 2006) and highlighting the importance of the NL in the organization and regulation of the genome during the aging process.

## Chromatin Organization in the Nuclear Space

In interphase, individual chromosomes cannot be visualized with simple phase contrast microscopy and it was first supposed that mitotic chromosomes rapidly entangle after decondensation. However, Rabl and Boveri demonstrated that plant chromosomes conserve polarity in interphase nuclei and hypothesized that chromosomes occupy discrete territories within the nucleus (Cremer et al., 1982; Rabl, 1885). This hypothesis was further demonstrated with the work of Cremer and collaborators who observed that UV laser irradiation of discrete regions of Chinese hamster cell nuclei damages only a small subset of mitotic chromosomes (Cremer et al., 1982). More recently, the use of whole chromosome painting probes revealed that individual chromosomes occupy distinct territories and that homologous Chromosomes Territories (CTs) are not adjacent (Cremer and Cremer, 2001). However, interchromosomal interactions are also observed between adjacent CTs and can be driven through association with nuclear substructures (Branco and Pombo, 2006; Lomvardas et al., 2006). More recently, the rise of next generation sequencing has brought further insights into the higher-order, organization of DNA territories and regulation of intra- or interchromosomal interactions (de Wit and de Laat, 2012).

At the molecular scale, chromatin is constantly moving by Brownian motion and chromatin flexibility allows the spatial relocation of genomic segments (Marshall et al., 1997; Edelmann et al., 2001; Gerlich et al., 2003). In many instances, the position of a particular sequence is correlated with its degree of compaction or transcriptional status, depends on the nature of nuclear substructures it interacts with and *cis* or *trans* chromatin interactions (Link et al., 2013). In budding yeast and mammalian cells, some regions can be tethered to subnuclear sites such as the nuclear or nucleolar periphery where chromatin motion is subsequently reduced while being more dynamic in the inner part of the nucleus (Heun et al., 2001; Chubb et al., 2002). Heterochromatin, gene-poor regions or switched-off genes are mostly localized within three intranuclear regions; the pericentromeric bodies, perinucleolar regions, and the NP (Lemaitre and Bickmore, 2015; Saksouk et al., 2015; Politz et al., 2016). Gene-poor CTs are usually found at the NP whereas gene-rich CTs are localized internally (Croft et al., 1999; Boyle et al., 2001), a spatial organization conserved in primates (Tanabe et al., 2002). Nevertheless, even if active regions rather lie within the inner nuclear space, positioning of genes at the NP, in the vicinity of the NPC is associated with active transcription in many organisms (Blobel, 1985; Ibarra and Hetzer, 2015; Ben-Yishay et al., 2016). *Cis*-looping of chromosomal regions represents the majority of chromatin–chromatin interactions. Nevertheless, increasing evidence for interchromosomal association accumulates and transcriptional regulation seems to be a major determinant in this process possibly through functional sequence redistribution (Fullwood et al., 2009; Lieberman-Aiden et al., 2009; Tang et al., 2015). Thus, the position of a gene within the nucleus is not random and inter- or intra-chromosomal interactions are often required for the coordinated regulation of loci but also for other DNA processes such as replication or DNA repair. In addition, plasticity of the chromatin fiber can be limited by formation of higher-order structures linked to interactions with the INM.

## The Nuclear Envelope Influences Nuclear Organization and Chromatin Regulation

Early studies on purified nuclear “shells” uncovered the presence of tightly packed chromatin stably associated to nuclear Lamins (Bouvier et al., 1985; Boulikas, 1986). Since, accumulation of evidences supports a key role for Lamins and INM components in chromatin organization. Among Lamins and INM proteins, many bind directly to chromatin or recruit intermediate factors. Lamins interact with naked DNA in a non sequence-specific manner (Shoeman and Traub, 1990; Stierle et al., 2003) but also to mitotic chromosomes (Glass et al., 1993). Amino acids 396–430 of the human Lamins A/C tail binds core histones *in vitro* (Taniura et al., 1995) and *in vivo* (Taniura et al., 1995; Goldberg et al., 1999; Mattout et al., 2007). At the level of DNA, Lamins bind to A-T rich sequences called scaffold/matrix attachment regions (S/MARs) *in vitro* depending on their polymerization state (Luderus et al., 1992, 1994; Zhao et al., 1996) and *in vivo* (Guelen et al., 2008). In addition, interactions with specific loci have been described in the mouse where Lamin-associated domain at the *IgH* and *Cyp3a* loci are enriched for repetitive GAGA sequences (Zullo et al., 2012) while, in human cells, the *D4Z4* macrosatellite elements is tethered to the lamina through interactions with a GA-rich motif (Ottaviani et al., 2009, 2010).

Besides direct interactions with DNA, both A- and B-type Lamins associate with chromatin through LEM-domain proteins (LAP2-Emerin-MAN1), particularly LAP2 isoforms (LAP2α and LAP2β), which in turn bind to BAF1. BAF1 is a critical chromatin-lamina bridging factor and expression of *BANF* mutants in human cells prevents the correct assembly of the nuclei after mitosis (Haraguchi et al., 2001). Last, the Lamin B Receptor (LBR) specifically interacts with B-type Lamins and binds to DNA and the Heterochromatin Protein 1 (HP1; **Figure 1**; Polioudaki et al., 2001).

The multiple layers of association between lamina-associated factors and chromatin thus constitute a very dense network at the NE serving as an anchoring platform for genome organization and chromatin interactions.

## The Nuclear Periphery As A Scaffold for the Folding of Large Chromosomal Domains

Evidences such as the mislocalization away from the NP of chromosome 13 and 18 in cells from patients carrying mutations in Emerin or A-type Lamins (Meaburn et al., 2007) linked the repartition of whole CTs with the lamina and suggested that integrity of the NL is essential for correct CTs organization (McCord et al., 2013).

**FIGURE 2:**
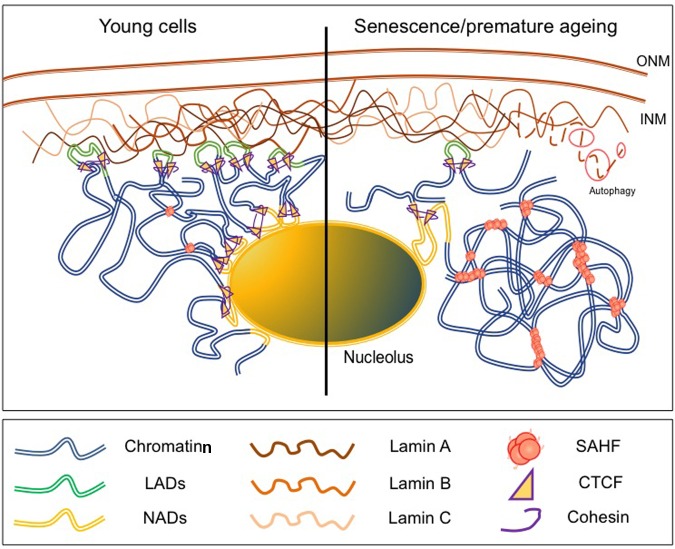
**Chromatin is rearranged upon physiological or premature aging.** **(Left)** In young cells, large chromatin domains [topologically associated domains(TADs), Lamin Associated Domains (LADs)] are associated to the NL and localized at the nuclear periphery (NP). These domains mainly correspond to repressed loci and are flanked by CTCF and Cohesin binding sites. **(Right)** In senescent cells or cells from laminopathy patients, chromatin organization is modified upon disruption of the lamina either after degradation of B-type Lamins by autophagy or production of dysfunctional A-type Lamins. LADs are relocalized away from the NP or the nucleolus and correlates with the formation of SAHF (Senescent Associated Heterochromatin Foci) enriched in repressive histone marks and chromatin remodeling factors such as HIRA, ASF1a, HP1 or MacroH2A.

Recent experimental developments provided new insights on the spatial organization of chromatin during interphase. Three- or four-dimensional light microscopy and cellular models using the tagging of loci *ex vivo* through the targeting of fluorescent fusion proteins allowed researchers to follow the spatial dynamics of a single locus in a single cell throughout cell cycle and cell differentiation. More recently, new techniques such as Chromatin Conformation Capture (3C; Dekker et al., 2002, 2013) and derivatives (4C, 5C, HiC), ChIA-PET (de Wit and de Laat, 2012), RNA Tagging and Recovery of Associated Proteins (RNA TRAP) and DamID (Dam IDentification) based on the ectopic production of a chimerical bacterial DNA adenine methyltransferase (Dam) fused to a chromatin protein (van Steensel and Henikoff, 2000; Guelen et al., 2008), have been powerful tools to detect inter- or intrachromosomal interactions and to decipher 3-dimensional genomic organization. The use of these different techniques has led to the identification and description of subdomains such as LADs (Lamin Associated Domains; Guelen et al., 2008) and NADs (Nucleolar Associated Domains; van Koningsbruggen et al., 2010) formed by association of chromatin with the NL and the nucleolus, respectively; while individual chromosomes are organized in active and open domains or inactive and condensed structures named TADs (topologically associated domains; Dixon et al., 2012; Nora et al., 2012; Sexton et al., 2012; Phillips-Cremins et al., 2013) (**Figure 2**). TADs are separated by boundary regions that contain CTCF binding sites or housekeeping genes able to block interactions between adjacent TADs. These TADs are conserved between cell types but contain smaller domains, or sub-TADs diferentially regulated depending on the cell type or differentiation stage (Phillips-Cremins et al., 2013; Rao et al., 2014). Recent evidences suggest a key role for TADs and TAD boundaries in a number of congenital diseases (Ibn-Salem et al., 2014). For instance, in a case of ADLD, a deletion upstream of the *LMNB1* gene eliminates the TAD and its boundary causing ectopic interactions between at least three forebrain enhancers and the *LMNB1* promoter leading to Lamin B1 overexpression, as observed in other typical ADLD cases (Brussino et al., 2010; Giorgio et al., 2015). Nevertheless, to date, nothing is known on the role of the NE in TADs regulation. We will only focus here on LADs and the role of the NL in the distribution and regulation of these chromatin domains.

Initial genome-wide mapping using the DamID technique in human cells has revealed the existence of about 1,100–1,400 LADs defined as large genomic regions closely associated with the lamina through interaction with Emerin or B1-type Lamin (Guelen et al., 2008; **Figure 2**). These 0.1–10 Mb gene-poor regions are characterized by weak expression and a repressive chromatin signature supporting the view of the NP as a heterochromatin-clustering compartment. Similar LADs were identified in mammalian cells using fusions with Lamin A, LBR and BAF (Guelen et al., 2008; Meuleman et al., 2013; Kind and van Steensel, 2014) possibly organized in two different complexes, one formed by Lamin B1 and LBR and the second one containing A-type Lamins and LEM domain proteins such as LAP-Emerin and MAN1 (Solovei et al., 2013). Interestingly, genome-wide studies have shown that NADs overlap with LADs, reinforcing the idea that repressed domains can shuttle between heterochromatic compartments such as the NP or the nucleolus (Nemeth et al., 2001; van Koningsbruggen et al., 2010). LADs can be relocated in the absence of Lamin B1, B2 or BAF while relocation of NADs mostly depends on BAF and Lamin A suggesting a dynamic regulation of the two types of topological structures by specific components of the NL (Kind and van Steensel, 2014; Padeken and Heun, 2014).

The NL is disassembled during mitosis and reassembled at the surface of chromosomes at the anaphase-telophase transition (Moir et al., 2000). Contacts between the NL and LADs are dynamic, established in a stochastic manner early after mitosis and depends on the cell-to-cell or allele-to-allele variation in the level of Histone H3 dimethylated Lysine 9 (H3K9me2) at the surface of the different LADs with a key role for the G9a histone methyltransferase in LADs formation and organization (Guelen et al., 2008; Peric-Hupkes and van Steensel, 2010; Kind and van Steensel, 2014). Nevertheless, little is known on how LADs are directed to the NL. Using an artificially tagging system, YY1 (Yin Yang 1) has recently been involved in LAD-tethering, dependent on histone H3 lysine 27 trimethylation and lysine 9 di- and trimethylation (Harr et al., 2015). YY1 might facilitate recruitment of PRC2 (Polycomb repressive complex 2) to LAD borders. In addition, Lamin C seems more specifically implicated in the repositioning of PRC2 to LADs borders compared to Lamin A (Harr et al., 2015). However, the precise orchestration of this process remains to be established.

Lamins directly interact with DNA, histones and contribute with Lamin binding proteins to the higher-order chromatin organization and gene regulation. It is thus not surprising that changes in the organization of the NL associated with normal and pathological aging profoundly impact genome topology, gene expression and genome stability as detailed below.

## Lamins and Lamin-Binding Proteins in Chromatin Organization

Lamins are required for the organization of chromatin at early developmental stages (Melcer et al., 2012). Absence of Lamins affects chromatin plasticity and the differentiation potential of embryonic stem cells (ESCs) while ectopic *LMNA* expression restricts the dynamics of heterochromatin proteins such as histone H1 (Melcer et al., 2012). LBR binds to H3K9me3 via Heterochomatin Protein 1 (HP1; Ye and Worman, 1996) and to H4K20me3 via its Tudor domain (Hirano et al., 2012; **Figure 1**).

Lamin A and the LBR protein might play similar functions but at different differentiation stages. LBR is predominantly expressed at early developemental stages while Lamin A is expressed later. LBR is sufficient to tether heterochromatin at the NP and LBR knock-down in Lamin A-deficient cells and *vice versa* induces inverted chromatin distribution with heterochromatin accumulation at the center of the nucleus and euchromatin at the periphery (Solovei et al., 2013).

The dynamics of nucleus reformation after mitosis further supports a major role for other NE-associated proteins in constraining and organizing the nuclear architecture. BAF and INM proteins (LBR, LAP2β, LAP2α, or Emerin) are associated with the early presence of NE vesicles around chromatin at late anaphase in HeLa cells, and segregate differently with chromatin during nuclear reformation (Haraguchi et al., 2000, 2001; Dechat et al., 2004). LAP2β and Emerin, two members of the LEM protein family that interact with chromatin through the LEM domain of the BAF protein and to DNA via its LEM-like domain (Cai et al., 2001) contribute to gene repression by interacting with the histone deacetylase 3 (HDAC3) and transcriptional repressors (Somech et al., 2005a,b; Demmerle et al., 2012; Zullo et al., 2012). Another LEM protein, LAP2α is a non-membrane associated isoform of the LAP2 protein family. LAP2α shares a common N-terminus with other LAP2 proteins but has a unique C terminus lacking a transmembrane domain (Dechat et al., 2000). LAP2α specifically interacts with A-type Lamins (Dechat et al., 2004) and the Retinoblastoma protein (Markiewicz et al., 2002; Dorner et al., 2006). Lap2α loss in mice is associated with defects in different tissues such as skin, colon, hematopoietic system, skeletal muscle and heart, and causes loss of Lamin A (Naetar et al., 2008; Gotic and Foisner, 2010; Gotic et al., 2010a,b). However, *Lap2*α knock-out *Lmna^-^*^/-^ rescues the muscular phenotype and extends life of the mice (Gotic et al., 2010b).

LAP2 binds to chromatin and telomeres and modulates association of the high mobility group protein N5 (HMGN5) to chromatin (Zhang et al., 2013). In addition, both A-type and B-type Lamins are required for the recruitment of factors involved in DNA repair (Butin-Israeli et al., 2013). Lamin B1 anchors proteins involved in nucleotide excision repair (Butin-Israeli et al., 2013). Furthermore, as recently described for telomeres, mobility of chromosome internal double strand breaks (DSB) is mediated by 53BP1/SUN1/2 [p53 Binding Protein 1/(Sad1p, UNC 84 proteins 1 and 2)] and microtubules (Lottersberger et al., 2015) opening new perspectives for understanding DNA damage response defect in laminopathies.

Lamins A/C are also found as soluble proteins in the nuclear interior. Using euchromatin- and heterochromatin-enriched samples, it has recently been shown that soluble Lamins A/C associates with euchromatin and that Lamins A/C-enriched fractions overlap with those bound by LAP2α (Gesson et al., 2016). On the other hand, Lamin B1 is mostly associated with heterochromatin and *Lap2α* deficiency shifts binding of Lamins A/C toward heterochromatin (Gesson et al., 2016). In premature aging syndromes, expression of progerin causes loss of nucleoplasmic Lamins A/C and LAP2α (Vidak et al., 2015).

## Transcriptional Control At the Nuclear Periphery

If the NP is generally associated with heterochromatin and silent chromatin modifications, causality between the repositioning of genes away from the NP and transcriptional activation is not always clear and a number of examples escapes this strict definition.

The movement of immunoglobulin loci away from the NE occurs in mouse pro-B cells, prior to recombination and transcriptional activation (Kosak et al., 2002), whereas robust expression of the murine β*-globin* gene can be detected while the gene is still associated with the NL, at stages preceding locus displacement (Ragoczy et al., 2006). Upon differentiation of mouse ES cells into neural progenitors, the *Mash1* locus is displaced away from the NE concomitantly with *Mash1* gene activation, however, expression of some neighboring genes is not affected (Williams et al., 2006). This displacement correlates with a low level of acetylated H3K9 and enhanced H3K27 methylation principally at the *Mash1* gene. Interestingly, ESCs mutated for histone and DNA methyltransferases (HMTs and DNMTs, respectively) do not exhibit changes in the association of the locus to the NP and it was suggested that histone acetylation rather than methylation could be functionally linked to the control of chromatin association to the nuclear rim. During mouse T-cell differentiation, the cell type-specific regulators of cytokine expression are repositioned away from the NE, whereas their target cytokine genes are not, such as the *IFN*-γ gene, constitutively associated to the NP even when transcriptionaly active in T helper types 1 and 2 (Th1 and Th2) cells (Hewitt et al., 2004). Finally, the human *CFTR* gene is also delocalized from the NP when activated. However, its delocalization after disruption of the peripheral heterochromatin with the HDAC inhibitor Trichostatine A (TSA) is not accompanied with transcriptional upregulation. On the other hand, inhibition of transcription with 5,6-dichlorobenzimidazole riboside (DRB) resulted in downregulation of active *CFTR* and a movement toward the NP (Zink et al., 2004).

In order to study the transcriptional and epigenetic modifications linked to changes in radial positioning, several research groups have developed methodologies based on the introduction of transgenes by artificially targeting fusion proteins to the NP in living cells. In the first one, using the *LacO* targeting system in mouse 3T3 cells, GFP and LacI were fused to the C-terminal end of Emerin allowing, upon removal of IPTG, the targeting of 70% of the transgenes to Lamins and Laps but not to NPC or pericentric heterochromatin (Reddy et al., 2008). Upon peripheral tethering, transgene expression and histone H4 acetylation are decreased. In human cells, similar results were described with a more complex system coupling independent targeting induction and transgene expression with simultaneous fluorescent labeling of the integrated construct, the transgene mRNA and protein (Kumaran and Spector, 2008). However, in this setting that couples LacI to Lamin B1, transgene expression is similar in terms of levels and polymerase recruitment whether it is targeted to the periphery or not. Nevertheless, both studies agreed that the relocation to the NP needs a cellular division to occur suggesting that the nucleus has to go through very plastic phases during mitosis and early G1 in order to efficiently achieve chromatin reorganization. One last study, using fusion to LAP2β for targeting and random integrations of *LacO* arrays in the human genome, reported a downregulation of genes neighboring the transgene upon targeting to the NE (Finlan et al., 2008). This silencing effect can be reversed by untethering the locus with IPTG treatment, and is also sensitive to TSA, even if the initial positioning remains. However, some neighboring genes escape silencing, suggesting that peripheral anchoring is not always sufficient to suppress transcriptional activity. On the contrary, targeting the transactivator domain of VP16 (Herpes Simplex Virus Protein vmw65) results in transgene stabilization in the interior of the nucleus and is dependent on nuclear actin and myosin suggesting the existence of active and specialized mechanisms compared to the passive repositioning described above (Chuang et al., 2006).

In conclusion, what emerges from these different studies and models is the possibility of peculiar situation with transcription occuring at the NP and silencing in the nuclear interior even if fewer cases are reported for the later.

## Lamina and Chromatin Remodeling During Normal and Pathological Aging

Lamin B1 is down-regulated in senescent cells and forced reduction of Lamin B1 leads to premature senescence, modifies the distribution of histone marks with a profound redistribution in H3K27me3-enriched or depleted regions (Shimi et al., 2011; Freund et al., 2012). In addition, changes in Lamin B1 contributes to SADS (senescence-associated distension of satellites), a large-scale decondensation of pericentromeric satellites (Swanson et al., 2013). In senescent cells, Lamin B1 decrease is mediated by autophagy via interactions between Lamin B1 and the LC3II autophagy marker at the NP (Dou et al., 2015) while Lamins A/C and B2 poorly bind to LC3 (**Figure 2**). Impressively, the lipidated form of LC3II overlaps with Lamin B1 binding sites and corresponds to heterochromatic LAD domains linking the autophagy machinery to the massive chromatin changes associated with senescence (Dou et al., 2015).

During replicative or oncogene-induced senescence, H3K4me3 at promoters of transcriptionally active genes and H3K27me3 at facultative heterochromatin display an altered distribution compared to proliferative cells with large domains either enriched in H3K4me3 or H3K27me3 (Sadaie et al., 2013; Shah et al., 2013). In proliferating cells, H3K4me3 enrichment is visible as sharp peaks at promoters after ChIP-Seq. In senescent cells, this euchromatin mark decorates extremely large domains spanning up to several hundred of kilobases corresponding to approximately 17% of the genome (Shah et al., 2013). H3K27me3 spans several hundred of kilobases in average and overlaps in a number of cases, with large H3K4me3-enriched loci forming large regions of bivalent chromatin while H3K27me3 is depleted in specific regions. Large regions enriched in H3K4me3 and H3K27me3 overlap with LADs while H3K27me3-depleted regions overlap with enhancers and form between LADs underlining the massive chromatin changes occurring during senescence and the close connection between these changes and the NL (**Figure 2**). Interestingly, a high proportion of genes encoding for proteins found in the senescence-associated secretory phenotype (SASP) are located in these large H3K27me3-depleted regions (Shah et al., 2013) strongly linking the integrity of the NE with the induction and propagation of the senescent phenotype.

In laminopathies, mutations in A-type Lamins alter chromatin regulation either by modifying directly the global organization, the interactions with chromatin-associated factors or by modifying the distribution of chromosomal domains with gene-poor regions relocated to the nuclear interior (Meaburn et al., 2007). In HGPS, progerin accumulation correlates with loss of heterochromatin, H3K9 trimethylation and decreased HP1 but increased H4K20me3 levels (Goldman et al., 2004; Misteli and Scaffidi, 2005; Shumaker et al., 2006; McCord et al., 2013) and correlates with reduction in the level of factors forming the Mi2/NURD complex that harbors both nucleosome remodeling and histone deacetylase activities, such as RBBP4, RBBP7, and HDAC1 (Pegoraro et al., 2009). Progerin expression induces decondensation of the inactive X chromosome (Shumaker et al., 2006) and satellite III transcription (Jolly et al., 2004; Shumaker et al., 2006). In HGPS, the global H3K27me3 level is decreased but not massively modified compared to senescent IMR90 cells while enrichment in H3K4me3 is observed over large regions (Shah et al., 2013). In mouse cardiac myocytes and embryonic fibroblasts expressing progerin, nearly all (99.5%) of lamina-associated genes attach to the INM (Kubben et al., 2012). Besides, progerin enhances interaction between the NL and a specific subset of additional genes suggesting specific function for this protein in the genomic dysregulation associated with aging. Progerin also sequesters the NRF2 [Nuclear Factor (erythroid-derived 2)-like 2] transcription factor impairing thereby the response to oxidative stress leading to an accumulation of ROS as well as an impairment of the DNA damage response, especially in mesenchymal stem cells (Kubben et al., 2016).

Farnesylation also influences Lamin-chromatin interactions. Non-farnesylated pre-lamin A recruits Lap2α, HP1α, and BAF1 into nuclear foci (Mattioli et al., 2008; Capanni et al., 2010) whereas farnesylated pre-lamin A interacts with NARF and SUN1 (Mattioli et al., 2011) but blocks interaction with HP1α. Thus, disruption of the NL might be perceived as a major stress for the cell with profound impact on global epigenetic regulation, chromatin marks spreading along large chromosomal domains and chromatin topology. Hence, the gradual reassociation of A-type Lamins concomitantly to reformation of heterochromatin domains in early G1 indicates that the NE is a major backbone for nuclear organization and gene regulation at the NP. Molecular evidences supporting a role for the NE in heterochromatin tethering and regulation also come from the high number of BAF binding partners among INM proteins and the complexes formed by B-type Lamins, LBR LAP2β, HP1, and the HDAC3 deacetylase (Somech et al., 2005b). Nevertheless, A- and B-type Lamins might form separate meshworks with a preferential association of B-type Lamins with silenced regions and A-type Lamins with euchromatin (Shimi et al., 2008).

Another HGPS-related *LMNA* mutation (E145K) in the central rod domain of Lamins A/C is associated with multilobulated nuclei and leads to alterations in pericentric chromatin, abnormal central clustering of centromeres, defects in the reassembly of the NE after mitosis or anaphase and mislocalization of telomeres (Taimen et al., 2009). In cells from a patient with atypical progeria, carrying a rare point missense mutation p.S143F (C428T) in the *LMNA* gene, blebs enriched in A-type Lamins but devoid of the major structural NE components have been observed (Bercht Pfleghaar et al., 2015). These blebs are vacant of centromeric heterochromatin and gene-poor regions but enriched in gene-rich chromosomal regions in which transcription is not globally inhibited or reduced. In MAD, *LMNA* mutation is associated with loss of heterochromatin or BAF, HP1β, and H3K9me3 mislocalization (Cenni et al., 2011; Capanni et al., 2012).

Similar defects were observed in mouse models of premature aging syndromes. The *Lmna*^Δ^*^8-11/^*^Δ^*^8-11^* mice express a truncated form of Lamin A lacking the domain encoded by exons 8–11 and develops abnormalities that resemble EDMD. MEFs from these mice present defects in non-homologous end joining (NHEJ) and homologous recombination (HR) together with alteration in telomere structure and function, with complete telomere loss (Gonzalez-Suarez et al., 2009; Redwood et al., 2011a,b) associated with a marked decrease in H4K20me3 at telomeres and pericentromeres.

Mutations in exon 9 of the *LMNA* gene are linked to HGPS (5527C) and muscular dystrophy (L530P). Interestingly, *Lmna*^Δ^*^9/^*^Δ^*^9^* mice display progeria features with faster telomere attrition compared to the *Lmna*^Δ^*^8-11/^*^Δ^*^8-11^* mice and chromatin defects but not genomic instability (Das et al., 2013). Interestingly, chromatin alterations mainly affect telomere and are not observed at pericentromeric regions suggesting that specific mutations have different impact on the epigenetic status of different heterochromatin compartments. However, the Δ*9/*Δ*9* mutation modifies the organization of pericentric chromatin suggesting a role for the domain encoded by exon 9 in the nuclear compartimentalization of heterochromatin (Das et al., 2013).

As observed in premature aging syndromes associated with defects of the NE, pre-Lamin A accumulates with normal aging and correlates with heterochromatin decondensation and recruitment of DNA repair factors (Scaffidi and Misteli, 2006). In addition, normal aging is accompanied with Lamin B1 downregulation and degradation by autophagy (Dou et al., 2015) underlining the key role of the NE in the occurrence of epigenetic changes associated with onset and progression of cellular senescence and organismal aging.

## Senescence-Associated Heterochromatin Foci, Chromatin Signature and Topology

As described above, senescence is associated with global changes in chromatin structure spatial heterochromatin rearrangement and decreased peripheral heterochromatin thickness (De Cecco et al., 2013; Sadaie et al., 2013). Senescent cells are also characterized by decreased repression of genes regulated by H3K27me3 associated with deficiency in the polycomb-associated EZH2 histone methyltransferase (Bracken et al., 2007). During aging, many mammalian cell types develop regions of condensed chromatin named Senescence-Associated Heterochromatin Foci (SAHF). SAHFs do not form in all senescent cells. Their production might depend on the senescence induction and are mainly associated with oncogenic senescence rather than replicative senescence (Narita et al., 2003; Zhang et al., 2005; Funayama et al., 2006; Jeanblanc et al., 2012).

Senescence-Associated Heterochromatin Focis are highly organized structures characterized by HP1 and H3K9me3 accumulation and composed of multiple concentric layers of chromatin where H3K9me3 forms a core surrounded by a layer of H3K27me3 (Chandra et al., 2012; Chandra and Narita, 2013). In oncogene-induced senescence, heterochromatin thickness is decreased at the NP and correlates with repositioning of heterochromatin marks to SAHF (Chandra and Narita, 2013; **Figure 2**). SAHFs control gene expression, especially of senescence-associated cell cycle arrest genes through their spatial repositioning to repressive regions (Narita et al., 2003; Zhang et al., 2007) or by suppressing the DNA damage response upon oncogene-induced senescence (Di Micco et al., 2011). H3K9me3-enriched chromatin domains such as pericentromeres or telomeres are not part of SAHF but localized at their periphery (Funayama et al., 2006; Narita et al., 2003, 2006; Zhang et al., 2007). Chromosome painting analyses showed that individual chromosomes form individual SAHF suggesting that these structures form at specific domains (Narita et al., 2003; Funayama et al., 2006; Ye et al., 2007; Zhang et al., 2007).

In addition to the two well-known HP1 and H3K9me3 heterochromatin components, other proteins contribute to this massive heterochomatinization such as MacroH2A, a histone variant associated with gene silencing and the HIRA (histone cell cycle defective homolog A) or ASF1 (anti-silencing function 1A) histone chaperones (Funayama and Ishikawa, 2007; Zhang and Adams, 2007; Zhang et al., 2007). When cells enter senescence, HIRA and the other members of the HUCA complex, CABIN1 and UBN1, localizes in PML (ProMyelocytic Leukemia) nuclear bodies (PML-NBs) and this process is required for SAHF formation (Zhang et al., 2005; Ye et al., 2007; Banumathy et al., 2009; Rai et al., 2011). Euchromatin marks are excluded from SAHF, including the H3.3 histone variant deposited by the HIRA chaperone.

HP1 proteins transiently localize to PML-NBs prior to their incorporation into SAHF. However, H3K9me3 and HP1 appear dispensable for the formation of the compacted SAHFs core while the HMG1A (High Mobility Group Protein 1A) that binds the AT-rich minor DNA groove is required for SAHF establishment and maintenance, possibly by bending DNA and promoting chromatin condensation (Narita et al., 2006). In addition, during senescence, Lamin B1 depletion from the central regions of LADs facilitates the repositioning of H3K9me3-rich regions toward the inner nuclear space and SAHF formation (Sadaie et al., 2013; Shah et al., 2013).

The absence of nascent RNA from SAHF indicates that these subnuclear domains mostly correlate with repressed regions (Narita et al., 2003; Funayama et al., 2006). The direct link between gene repositioning and regulation remains to be established but formation of these compact chromatin foci are thought to silence expression of genes such as those controlling the E2F, p16INK4a-RB and p53-RB pathways by producing a repressive environment that prevents transcription (Narita et al., 2006).

Although cells from HGPS patients show a decrease of heterochromatin but are devoid of SAHFs (Scaffidi and Misteli, 2006; Shumaker et al., 2006), recent findings uncovered similarities between SAHF- and progerin-induced senescence with the loss of contacts of GC-poor LADs prior to SAHF formation suggesting a two-step mechanism for SAHF formation involving also disruption of the NL (Chandra et al., 2015).

In agreement with an overall role for SAHF during the aging process, increased heterochromatinization and activation of the HIRA/ASF1A pathway have also been observed in different tissues and species and elderly people show increased heterochromatinization accompanied by transcriptional inactivation (Sarg et al., 2002; Herbig et al., 2006; Jeyapalan et al., 2007). Hence, chromatin remodeling observed in senescent cells likely reflects the modifications of the nuclear architecture also observed at the organismal level during physiological aging.

## Regulation of Human Telomeres At the Nuclear Periphery

Telomeres are nucleoproteic structures composed of repetitions of a T_2_AG_3_ motif combined to a protein complex called Shelterin mainly composed of six proteins [e.g., TRF1 (Telomeric repeat binding factor 1), TRF2 (Telomeric repeat binding factor 2), POT1 (Protection of Telomere 1), RAP1 (Repressor/Activator Protein 1), TPP1 (TINT1, PTOP, PIP1) and TIN2 (TRF1- and TRF2-Interacting Nuclear Protein 2)]. More particularly, TRF1 and TRF2 are found all along the T_2_AG_3_ repeats and are the main actors in the recruitment of the others (Palm and de Lange, 2008). The shelterin complex, located at the extremity of all chromosomes, ensures the stability of the genome and protects chromosome from the ectopic action of the DNA repair machinery (Palm and de Lange, 2008) by allowing the formation of a chromatin loop called T-Loop (Doksani et al., 2013). Due to the non-conservative replicative machinery, telomere shortens with each cell divisions and telomere erosion is seen in all human tissues (Daniali et al., 2013) making telomere size reduction one of the Hallmark of Aging (Lopez-Otin et al., 2013). Strikingly, among telomere-deficiency syndromes (or telomeropathies) such as Werner Syndrome (WS) and Dyskeratosis Congenita (DC) premature aging features are observed. WS is a recessive disorder caused by mutations in the *RECQL2* gene, causing the production of a truncated WRN protein, a DNA helicase involved in DNA repair (Moser et al., 2000a,b). Hence, WS individuals present signs of genomic instability, DNA repair, replication and apoptosis defects, along with susceptibility to cancer (Goto et al., 1996; Croteau et al., 2014). At telomeres, WRN facilitates replication by unwinding the shelterin structure and participates in the telomeric DNA damage regulation and processing (Crabbe et al., 2004, 2007; Eller et al., 2006). Another progeroid syndrome, DC is characterized by a failure of tissue homeostasis due to stem cell loss and renewal deficiency. Telomere dynamics is profoundly affected in DC due to haploinsufficiency of components of either the telomerase holoenzyme (*DKC1*, *TERT*, and *TERC*), the TCAB1 shuttling factor, SnRNP components (*NHP2* and *NOP10* required for telomerase activity), the TIN2 shelterin component or the regulator of telomerase length helicase, *RTEL1* (Heiss et al., 1998; Knight et al., 1999; Armanios et al., 2007; Savage, 2014).

Beyond the clear correlation between replicative senescence, telomere attrition and aging, an intriguing relationship also exists between telomeres and the NP. Progeria-associated *LMNA* mutation and progerin accumulation causes proliferation defect in primary fibrobalsts, telomere shortening and increased DNA damage response at telomeres, which can be rescued by *hTERT* expression (Allsopp et al., 1992; Kudlow et al., 2008; Decker et al., 2009). In addition, telomere erosion during replicative senescence induces progerin expression in normal fibroblasts (Cao et al., 2011). However, the direct link between telomere stability and the lamina remains partially understood. Earlier studies in human cells did not reveal any interaction between telomeres and the NL but with the nuclear matrix (de Lange, 1992). In the same line, another report on the localization of centromeres and telomeres described the peripheral clustering of mouse telomeres and centromeres whereas human telomeres localize in the nuclear interior (Weierich et al., 2003), except at meiosis where the LINC complex and SUN proteins contribute to the bouquet formation and the clustering of telomeres in a single subnuclear area with the pairing of homologous chromosomes (Penkner et al., 2009; Ding et al., 2007; Lottersberger et al., 2015). Nevertheless over the recent years, a number of reports revealed different links between telomere positioning or regulation and the NL. In particular, emerging evidences suggest that loss of A-type Lamins has an impact on telomere biology especially in progeroid syndromes (Decker et al., 2009). Some insights on the outcome of telomere positioning in cells invalidated for A-type Lamins were also obtained in mouse cells (Gonzalez-Suarez et al., 2009). Loss of A-type Lamins alters the subnuclear positioning of telomeres leading to telomere shortening, defect in telomeric heterochromatin, increased 53BP1-dependent genomic instability and hinders the processing of dysfunctional telomeres by NHEJ (Gonzalez-Suarez et al., 2009).

In human cells, dysfunctional telomeres linked to loss of the TRF2 or TIN2 shelterin proteins, travel greater distances than functional telomeres (Dimitrova et al., 2008; Chen et al., 2013). This mobility is dependent on 53BP1 and mediated by the SUN1 and SUN2 LINC complex proteins and connected to the cytoskeleton in particular through associations with Nesprin 4 and Kinesin 1 and 2 (Lottersberger et al., 2015). This 53BP1 and LINC complex-dependent mobility of dysfunctional telomeres promotes NHEJ and DNA repair suggesting a key role for the lamina-associated proteins in the subnuclear positioning of telomeres either during meiosis or in the DNA damage repair process (Lottersberger et al., 2015). Lamins A/C might also regulate telomeres directly through interactions with TRF2 while progerin do not. This interaction might contribute to the organization of long-range chromatin loops forming at interstitial telomeres encompassing megabases of chromatin, which might be disrupted in Lamin A/C deficient cells (Wood et al., 2014).

Telomeres are also regulated by a number of lamina-associated proteins. LAP2α forms discrete foci distributed throughout the nucleoplasm with many foci colocalized with telomeres (Chojnowski et al., 2015). At the end of mitosis, LAP2α and BAF stably associate with telomeres on one side of the decondensing chromatin during NE reformation, but then co-segregate with telomeres in the inner nuclear space (Dechat et al., 2004). LAP2α-telomeres association is impaired in HGPS (Chojnowski et al., 2015). LAP1, another type II transmembrane protein of the inner nuclear membrane is involved in the positioning of Lamins and chromatin might also associate with telomere through interaction with TRF2 and RIF1. However, the precise mechanisms linking LAP1, chromatin and telomeres remains to be uncovered (Serrano et al., 2016). In addition, Lamins-TRF2 interactions might stabilize t-loops forming at interstitial telomeric sequences (ITS) while progerin does not suggesting a key role for TRF2-Lamins interactions in the stability of telomeric sequences at chromosome ends and ITS (Wood et al., 2014).

Interestingly, in human cells, early replicating telomere are localized in the inner nuclear volume while peripheral positioning of telomeres correlates with late replication suggesting that components of the INM also control other aspects of telomere regulation (Arnoult et al., 2010) but this regulation might not be directly associated with the tip of chromosomes but with the subterminal subtelomeric regions. Telomeres are separated from chromosome-specific gene-rich region by complex sequences named subtelomeres. In the human population, these subtelomeric regions are highly polymorphic and their recombination rate is higher than in the rest of the genome. If the 46 human telomeres share the same structure, this subtelomeric unique arm-specific composition concurs to the propensity of telomeres to regulate gene expression but also to the replication and maintenance of chromosome ends and likely their subnuclear positioning (Ottaviani et al., 2008; Arnoult et al., 2010). These subtelomeric regions are associated with genome evolution and contribute to the regulation of telomeric position effect (TPE) and production of telomeric repeat contanning-RNA (TERRA) transcribed from the subtelomeric regions through the adjacent telomere. The implication of TPE and TERRA has been evoked in many diseases but rarely investigated in depth (Ballif et al., 2007; Shao et al., 2008; Maicher et al., 2012).

**FIGURE 3:**
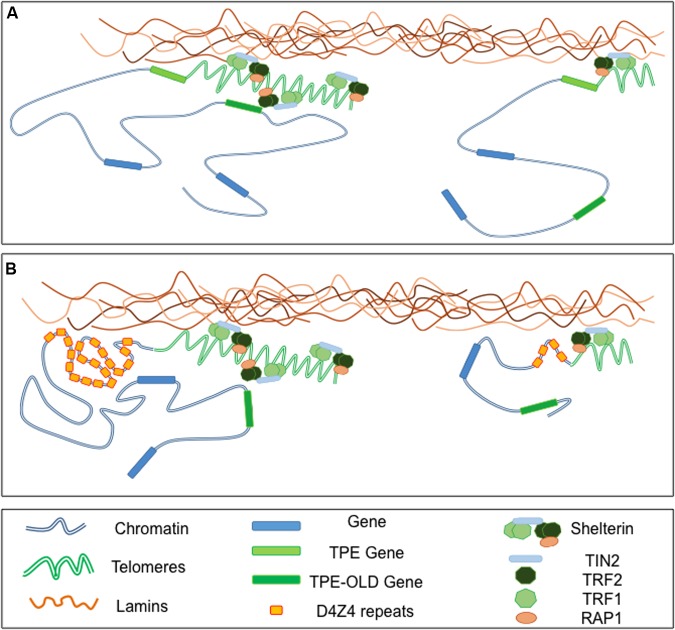
**Telomeres-Lamins interaction at the NP.** **(A)** In cells with long telomeres, Lamins A/C interact with TRF2, thus tethering some telomeres in a peripheral localization. Telomeres might also interact with soluble Lamins in the nucleoplasm. The silencing signature of telomeres can spread in *cis* (TPE-sensitive genes) but also in *trans* (TPE-OLD-sensitive genes) due to a mechanism yet to explore. With telomeres shortening, the diminution of repressive mark (TPE) or a local change in the chromatin conformation (TPE-OLD), modulate expression of TPE and TPE-OLD genes. **(B)** Both TPE and TPE-OLD have been described in FSHD, a muscular dystrophy genetically linked to the subtelomeric 4q35 locus. This subtelomeric locus and its abutting telomere are located at the NP and regulated by A-type Lamins. Moreover, the number of D4Z4 repeats influences the localization of the telomeres within the nuclei. In cells with few D4Z4 repeats the transcription of *DUX4*, a gene encoded by *D4Z4* repeats, is increased upon telomere shortening through direct TPE while expression of *SORBS2*, located 5 Mb upstream is up-regulated through a TPE-OLD phenomenon.

Overall, telomere positioning to the NP is variable between species and might be determined by subtelomere composition, although this remains to be determined for most human subtelomeres. Thus, transcriptional regulation of natural subtelomeric genes in human cells likely depends on telomere length, the structure of the telomeric chromatin but also on the composition of the subtelomeric regions and spatial distribution of chromosome ends. In addition, as observed experimentally using artificial systems (Baur et al., 2001; Koering et al., 2002), age-dependent telomere erosion might also be a key player in the modulation of subtelomeric genes in elders and contribute to physiological and pathological aging. In this model, if with long telomeres, a subtelomeric gene is captured within the structure, hence silenced, telomere shortening might unlock the gene from the heterochromatin signature and trigger gene derepression. Thus, over time telomere shortening can modify *in cis* or *trans* expression of various genes. In classical TPE, the degree of repression declines with distance from the telomere and spreading of silencing marks from the telomere might be limited to 100 kb in mammalian cells (Kulkarni et al., 2010). It was recently shown that telomeres affect gene expression over much larger distances through a telomere-length dependent looping (Robin et al., 2014). This phenomenon named TPE-OLD (TPE over long distance) can affect expression of genes located at a distance of up to 10 Mb from the telomere (**Figure 3A**). Unlike the classical TPE signature, this phenomenon is not continuous and relies in changes of the chromatin conformation. Interestingly, TPE-OLD also occurs in pathologies and was first described in Facio-Scapulo-Humeral Dystrophy, an age-dependent muscular dystrophy linked to the 4q subtelomere located at the NP and bound by A-type Lamins (Masny et al., 2004; Tam et al., 2004; Ottaviani et al., 2009, 2010; Arnoult et al., 2010; Robin et al., 2015) (**Figure 3B**). The role of telomere silencing by TPE or TPE-OLD, especially in pathologies is still in infancy (Ottaviani et al., 2008; Lou et al., 2009; Robin et al., 2014, 2015). However, converging evidence suggests that factors affecting directly or indirectly telomere length and structure will likely affect the topology and expression of subtelomeric regions. In this scenario, alterations of the lamina and production of progerin might interfere with telomere and subtelomere homeostasis and contribute directly or indirectly to diseases linked to these complex chromosomal regions as exemplified in FSHD.

## Conclusion

We highlighted here the diverse function of a number of proteins implicated in the filamentous network forming the NE. Some of them interact directly with chromatin, regulate chromatin architecture and organize the topology of chromosomal domains. In such a scenario, mutations in Lamins A/C or genes encoding other INM-associated factors in diseases and decrease in Lamin B1 upon senesecence contribute to physiological or pathological aging at different levels. Moreover, even if anchoring of genes at the NL might contribute to gene repression as suggested by artificial tethering systems (Finlan et al., 2008; Reddy et al., 2008; Dialynas et al., 2010), the mechanim of NL-mediated repression still remains to be elucidated. With a limited number of INM proteins characterized to date and our current knowledge of cell-specific variations in NE structures, the regulatory potential of the NP is a promising field of investigation in particular regarding the specificity of the phenotypes in the different types of nuclear envelopathies.

## Author Contributions

Both authors have contributed equally to the writing of the manuscript, edition and preparation of the figures.

## Conflict of Interest Statement

The authors declare that the research was conducted in the absence of any commercial or financial relationships that could be construed as a potential conflict of interest.
